# Worse prognosis for breast cancer diagnosed in advanced pregnancy and shortly postpartum: an update of the Dutch pregnancy-associated breast cancer cohort

**DOI:** 10.1007/s10549-025-07806-3

**Published:** 2025-08-12

**Authors:** Carsten F. J. Bakhuis, Stefan Preković, Britt B. M. Suelmann, Janneke Verloop, Pieter J. Westenend, Sabine C. Linn, Paul J. van Diest, Elsken van der Wall, Carmen van Dooijeweert

**Affiliations:** 1 Department of Pathology, University Medical Center Utrecht, Utrecht, The Netherlands; 2 Department of Medical Oncology, University Medical Center Utrecht, Utrecht, The Netherlands; 3 Center for Molecular Medicine, University Medical Center Utrecht, Utrecht, The Netherlands; 4 Netherlands Comprehensive Cancer Organization (IKNL), Utrecht, The Netherlands; 5 PALDordrecht, Dordrecht, The Netherlands; 6 Department of Medical Oncology, Netherlands Cancer Institute (NKI/AvL), Amsterdam, The Netherlands

**Keywords:** Breast cancer, Pregnancy, Postpartum, Miscarriage, Prognosis

## Abstract

**Purpose:**

Breast cancer diagnosed during pregnancy (PrBC) or postpartum (PPBC) is associated with a poorer prognosis, and earlier research indicated that outcomes differ based on timing of diagnosis. We updated and expanded our Dutch nationwide pregnancy-associated breast cancer (PABC) cohort, now also including patients diagnosed within one year after an interrupted pregnancy (AABC), to compare disease characteristics and prognosis across PrBC-, PPBC- and AABC subgroups and to non-PABC patients.

**Methods:**

All breast cancer pathology reports of women < 45 years in the Netherlands (1988–2022) were screened to identify patients diagnosed with PrBC, PPBC (< 12 months postpartum) or AABC (< 12 months after pregnancy interruption). PABC patients were 1:3 matched on age and year of diagnosis to non-PABC breast cancer patients.

**Results:**

In our PABC cohort (N = 787), the majority was diagnosed during pregnancy (n = 471, 60%). Two distinct prognostic subgroups were observed: a favorable group including trimester 1 PrBC, PPBC 6–12 months postpartum and AABC, and an unfavorable group diagnosed later in pregnancy (trimesters 2 and 3) or shortly postpartum (< 6 months). PABC patients showed overall, in comparison to non-PABC controls, poorer histopathological characteristics (more grade III and triple negative tumors) and a significantly worse 5-year overall survival (77% vs. 85%), persisting in multivariable analysis (HR 1.6, 95% CI 1.06 – 2.33, *P* = 0.025).

**Conclusions:**

PABC patients diagnosed in advanced pregnancy or shortly postpartum are most at risk for aggressive histopathology and an unfavorable prognosis. This highlights the need for in-depth analyses between specific PABC subgroups to elucidate the etiologic mechanisms involved.

**Supplementary Information:**

The online version contains supplementary material available at 10.1007/s10549-025-07806-3.

## Introduction

The incidence of breast cancer is rising, both in general as for young women (< 50 years), especially in countries in the highest level of the human development index. [1] Simultaneously, a rising incidence is observed for pregnancy-associated breast cancer (PABC). [2] This is likely due to the abovementioned rising incidence of breast cancer in young women as well as the increasingly older age at first pregnancy. [2, 3] However, standardized screening at childbearing age for patients at high risk for developing breast cancer, and the introduction of non-invasive prenatal testing which in rare cases is indicative of a maternal malignancy, may also contribute to a higher PABC-incidence. [3–5]

PABC is nowadays subdivided into breast cancer diagnosed during pregnancy (PrBC) and postpartum (PPBC). [6] The maximum duration of this postpartum interval differs between studies, with a meta-analysis observing a worse prognosis for PPBC patients diagnosed until six years after childbirth. [7] In general, PABC is often characterized by more aggressive disease characteristics, such as higher tumor grade, more frequent triple negative disease and a higher disease stage at diagnosis. [8–13] This translates into a worse prognosis for PABC patients, which seems to be caused mainly by these aggressive disease characteristics, as their prognosis in many studies is similar to non-PABC patients after adjustment for confounding variables. [7, 14, 15] In contrast, several meta-analyses and cohort studies did observe an independent worse prognosis for PPBC, especially for patients with a diagnosis shortly postpartum. [7, 16] The observed decrease in survival rates has primarily been attributed to the PPBC subgroup, in whom postpartum involution may play a crucial role in tumorigenesis. [14, 17] Nevertheless, several recent studies specifically examining trimester-specific subgroups of PrBC, reported more unfavorable histopathologic features and poorer prognosis in cases diagnosed during the second and third gestational trimesters, suggesting that adverse outcomes are not restricted to PPBC. [18–22] These suggested differences in tumor biology and outcome between the trimesters are especially interesting given the physiological variation during pregnancy trimesters in, amongst others, serum hormonal levels and immune cell functioning. [23, 24]

To further clarify the prognostic differences between PrBC and PPBC, we updated our nationwide Dutch Pregnancy-Associated Breast Cancer (DPABC) cohort [18], significantly increasing the number of included patients. Next to the updated follow-up (+ 3 years) and additional inclusion years (2020–2022) we have now included PPBC patients diagnosed up to one year postpartum (previously maximum 6 months) and patients diagnosed after an interrupted pregnancy (i.e., within one year after a spontaneous or induced abortion, abbreviated as AABC (“after abortion breast cancer”)). In this study, we specifically compare disease characteristics and maternal outcomes for each gestational trimester (for PrBC), specific time intervals for the PPBC and the AABC subgroup in general, and compare these to matched non-PABC controls.

## Materials and methods

### Data collection of PABC cohort

**Fig. 1:**
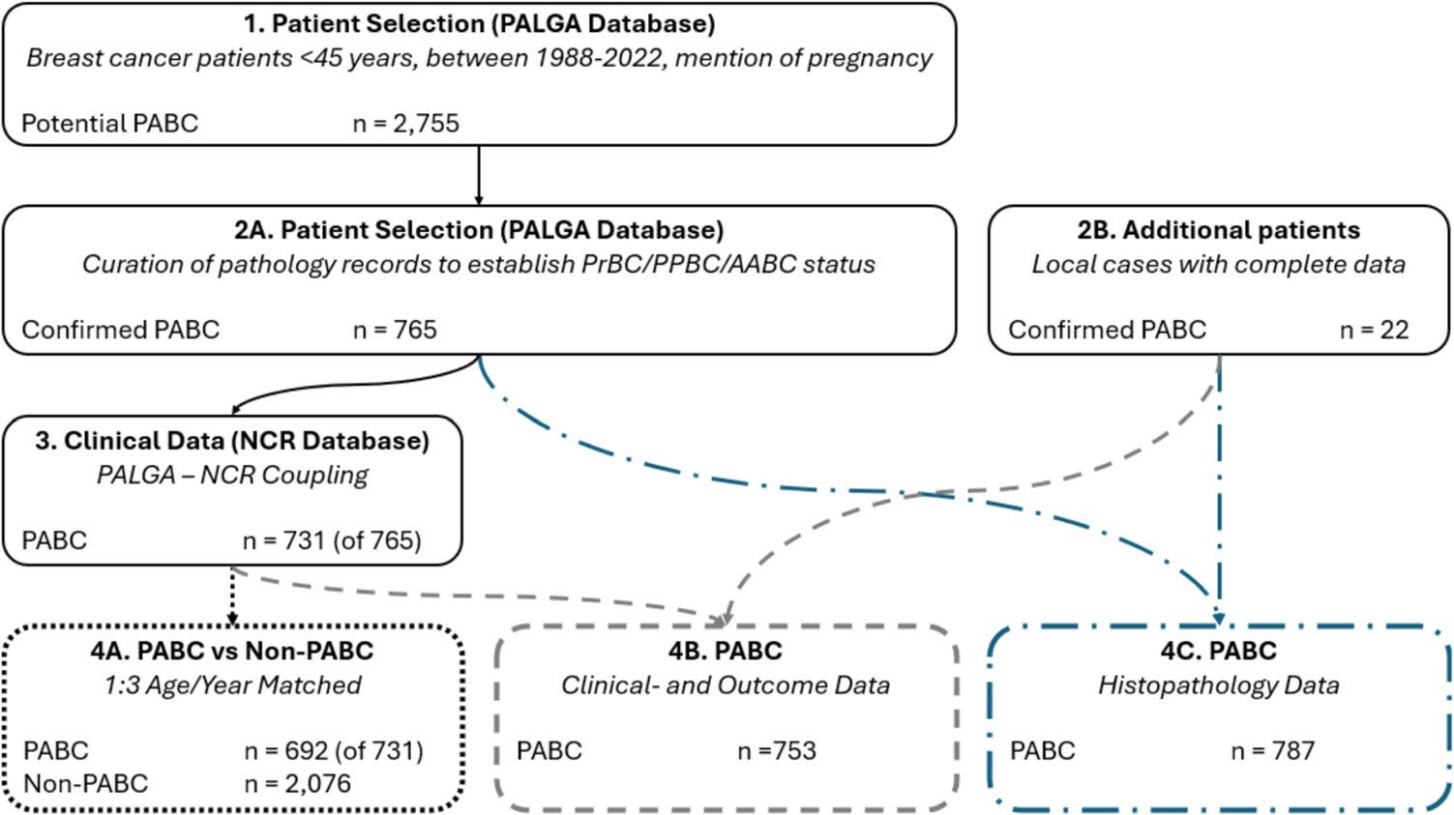
Flowchart displaying the selection of the database, coupling to the Netherlands Cancer Registry (NCR) and following 1:3 matching to a non-PABC cohort *PABC Pregnancy-Associated Breast Cancer, PrBC Breast cancer during pregnancy, PPBC postpartum breast cancer, AABC breast cancer after interrupted pregnancy, PALGA the Dutch Nationwide Network and Registry for Histo- and Cytopathology, NCR Netherlands Cancer Registry*

The Dutch nationwide network and registry for histo- and cytopathology (PALGA) was again utilized to compile our cohort. All pathology reports of women (18–45 years old) between January 1 st 1988 and July 1 st 2022 diagnosed with invasive breast cancer were screened for mentioning of at least one of several predefined pregnancy-associated keywords (e.g., “pregnancy”, “gestation”, “amenorrhea duration”, “lactation” et cetera). In addition, patients with a pathology report of either a complete placenta or endometrial curettage within one year of their breast cancer diagnosis were included for screening. Altogether, this resulted in 8,849 pathology reports from 2,735 patients (Fig. 1, step 1). These records were manually screened to include all patients with a diagnosis of invasive breast cancer during pregnancy (PrBC), within 12 months postpartum (PPBC) or within 12 months after a known interrupted pregnancy (AABC, either spontaneous or induced abortions). This resulted in a database of 765 patients (Fig. 1, step 2 A). Twenty-two additional cases were provided from a local series of pregnancy-associated breast cancer patients (Fig. 1, step 2B), resulting in a baseline dataset of 787 PABC-patients (including PrBC/PPBC/AABC, cohort 4 C in Fig. 1). Three included patients were older than 45 years at diagnosis. These patients were identified in the initial 8,849 reports because they had a pregnancy-related pathology report (usually a placenta or curettage) between 18 and 45 years from a previous pregnancy, but developed breast cancer during or after another pregnancy at an older age.

 For all included patients, baseline characteristics, pregnancy-related data and histopathological data were extracted from pathology reports. PrBC cases were subdivided by timing of their diagnosis in the first (gestational age of 1–12 weeks), second (weeks 13–26) or third (≥ 27 weeks) trimester. For one group of PrBC patients, although their concurrent breast cancer diagnosis and pregnancy could be confirmed, we were not able to determine the definitive trimester of diagnosis, and this group was subsequently lumped as “trimester unknown”.

Pregnancy outcomes (either live birth, spontaneous- or induced abortion) for the PrBC patients were derived from clinical data in subsequent pathology reports, for example when a placenta or fetal tissue (e.g., after curettage) was submitted for histological analysis.

The PABC-patients who were included from the PALGA database (n = 765) were then linked to the Dutch Cancer Registry (NCR), maintained by the Netherlands Comprehensive Cancer Organization (IKNL). Using the NCR, clinical and maternal outcome data was retrieved, which was successful for 731 PABC patients (Fig. 1, step 3). The maternal outcome data in the NCR is derived through a linkage to the Personal Records Database (BRP) of the Netherlands, with February 1 st 2023 as the index date for survival or death. Together with the previously mentioned local series of 22 patients with complete histopathological and clinical data that was obtained from a single pathology laboratory apart from the PALGA and NCR linkage, this resulted in histopathological and clinical data on a total of 753 patients (Fig. 1, cohort 4B).

### Control non-PABC cohort

Through the NCR, a control cohort was assembled. PABC patients were matched for age and year of diagnosis, where each PABC patient was matched to three unique individual non-PABC comparators. This matching was possible for 692 PABC patients, resulting in a control cohort of 2,076 matched non-PABC patients (Fig. 1, cohort 4 A).

### Data extraction

For the PABC cohort, all pathology records were available through PALGA. For these patients, detailed tumor characteristics were extracted from the pathology reports, including histological subtype, Bloom & Richardson grade, estrogen receptor (ER) status, progesterone receptor (PR) status and human epidermal growth factor receptor 2 (HER2) expression. In case of bilateral or multiple primary tumors, data on the tumor with the highest Bloom & Richardson grade or (when equal) the largest tumor diameter was used. For the non-PABC comparators, only tumor- and clinical characteristics registered in the NCR were available.

### Statistical analysis

**Fig. 2:**
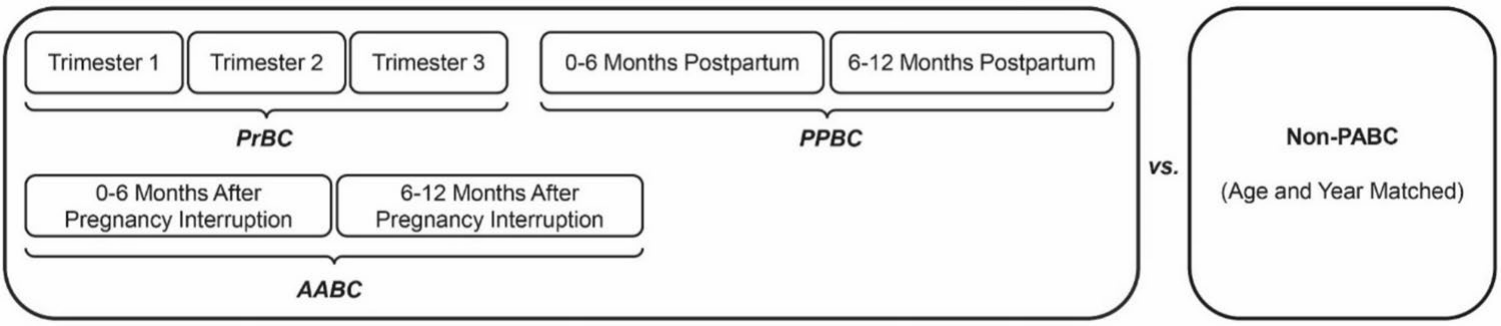
Overview displaying the various subgroups within our PABC cohort (PrBC per trimester, and PPBC/AABC per six month intervals) and the comparison to non-PABC control patients *PABC Pregnancy-Associated Breast Cancer, PrBC Breast cancer during pregnancy, PPBC postpartum breast cancer, AABC breast cancer after interrupted pregnancy*

As displayed in Fig. 2, histopathological and clinical data were compared between PABC and non-PABC and within PABC between the subgroups (PrBC, PPBC and AABC). For univariable comparisons, either Pearson Chi-Square with Yates’ continuity correction (all frequencies ≥ 5) or Fisher’s Exact tests (at least one frequency < 5) were used. For overall survival (OS) we calculated the time from initial diagnosis to the time of death or censoring date (i.e. February 1 st, 2023). To limit potential survivor bias for statistical modelling, patients were censored after 10 years of follow-up (3,653 days). All survival analyses were performed using the logrank (Mantel-Cox) procedure.

In the multivariable analysis, a Cox regression model was used (stratified for cases and their matched controls). P-values of < 0.05 were considered statistically significant, and were not corrected for potential multiplicity of testing.

 All statistical analyses were performed using R version 4.3.1 (R Foundation for Statistical Computing, Vienna, Austria), using the R packages *survival* (v3.8–3), *survminer* (v0.5.0), *gtsummary* (v2.1.0), *stats* (v4.4.2) and *ggplot2* (v3.5.1).

### Ethical approval

This study used available registry data from both PALGA and the NCR, and was approved by the respective scientific and privacy committees of both organizations. The pseudonymized data linkage between these databases was performed by a trusted third party (ZorgTTP, Houten, the Netherlands). In addition, this study was evaluated by our institutional review board (decision no. 19/366) which waived the need for ethical approval as the study does not fall under the scope of the Dutch Medical Research Involving Human Subjects Act (WMO).

## Results

Our updated cohort with available histopathological data now comprises of 787 PABC patients, of whom 753 (96%) had clinical and outcome data available. We matched each of the 692 PABC patients to three non-PABC control patients, yielding a total of 2,076 non-PABC controls.

### *PABC baseline (N* = *787 histopathology data, n* = *753 clinical/outcome data)*

The majority of PABC-patients were diagnosed during pregnancy (PrBC: 471 patients, 60%), followed by postpartum diagnosis (PPBC: 202 patients, 26%) and after an interrupted pregnancy (AABC: 114 patients, 15%). Median age at diagnosis across all PABC-subgroups was similar and ranged between 34 and 36 years. A detailed description of histopathological and clinical characteristics of the overall PABC cohort and the individual PrBC, PPBC and AABC subgroups is shown in Table 1. Of the 97 PrBC patients diagnosed in the first trimester, the pregnancy outcome was certain of 70 patients. Of them, 29 patients (41%) had an interrupted pregnancy (either spontaneous or induced) after diagnosis. When evaluating the time to first treatment (either surgery or systemic neoadjuvant treatment), known for 598 patients, this ranged between 16 days for patients diagnosed in the third trimester of pregnancy to 26 days for patients diagnosed in the first trimester.

**Table 1:** Comparison of histopathologic and clinical characteristics within the pregnancy-associated breast cancer cohort by timing of diagnosis: during pregnancy (PrBC, n = 471), within one year postpartum (PPBC, n = 202) or within one year after an interrupted pregnancy (AABC, n = 114)

		PABC Overall	During Pregnancy PrBC				** < **1Y Postpartum PPBC		** < **1Y After Interrupted Pregnancy AABC	
		PrBC/PPBC/AABC	Trimester 1	Trimester 2	Trimester 3	Unknown	0–6 Months	6–12 Months	0–6 Months	6–12 Months
		(N = 787)	(n = 97)	(n = 106)	(n = 203)	(n = 65)	(n = 126)	(n = 76)	(n = 64)	(n = 50)
		N (%)	N (%)	N (%)	N (%)	N (%)	N (%)	N (%)	N (%)	N (%)
Median Age (years)		34	34	34	34	34	34	35	36	36
Histological Subtype										
	NST	616 (84%)	83 (89%)	87 (87%)	157 (82%)	47 (80%)	91 (82%)	55 (83%)	51 (84%)	45 (90%)
	Lobular	17 (2%)	2 (2%)	2 (2%)	6 (3%)	3 (5%)	1 (1%)	2 (3%)	1 (2%)	0 (0%)
	Other	98 (13%)	8 (9%)	11 (11%)	28 (15%)	9 (15%)	19 (17%)	9 (14%)	9 (15%)	5 (10%)
	Missing	56	4	6	12	6	15	10	3	0
B&R Grade										
	1	30 (5%)	8 (10%)	1 (1%)	4 (2%)	2 (4%)	6 (6%)	3 (5%)	6 (12%)	0 (0%)
	2	175 (27%)	25 (30%)	20 (21%)	44 (27%)	11 (23%)	23 (23%)	19 (30%)	14 (28%)	19 (50%)
	3	436 (68%)	51 (61%)	73 (78%)	115 (71%)	35 (73%)	71 (71%)	42 (66%)	30 (60%)	19 (50%)
	Missing	146	13	12	40	17	26	12	14	12
Estrogen Receptor										
	ER- (< 10%)	372 (53%)	32 (36%)	55 (57%)	106 (57%)	34 (61%)	72 (65%)	30 (44%)	24 (44%)	19 (42%)
	ER+ (≥ 10%)	334 (47%)	58 (64%)	42 (43%)	80 (43%)	22 (39%)	38 (35%)	38 (56%)	30 (56%)	26 (58%)
	Missing	81	7	9	17	9	16	8	10	5
Progesterone Receptor										
	PR- (< 10%)	407 (59%)	37 (42%)	59 (62%)	107 (59%)	36 (68%)	80 (73%)	35 (53%)	30 (60%)	23 (52%)
	PR+ (≥ 10%)	279 (41%)	51 (58%)	36 (38%)	74 (41%)	17 (32%)	29 (27%)	31 (47%)	20 (40%)	21 (48%)
	Missing	101	9	11	22	12	17	10	14	6
HER2 Status										
	HER2-	424 (72%)	51 (67%)	67 (77%)	113 (72%)	35 (74%)	63 (69%)	37 (64%)	29 (71%)	29 (83%)
	HER2+	169 (28%)	25 (33%)	20 (23%)	45 (28%)	12 (26%)	28 (31%)	21 (36%)	12 (29%)	6 (17%)
	Missing	194	21	19	45	18	35	18	23	15
Intrinsic subtypes										
	HR+ HER2-	200 (34%)	35 (46%)	28 (33%)	49 (31%)	12 (26%)	25 (27%)	21 (37%)	14 (36%)	16 (47%)
	HR+ HER2+	101 (17%)	14 (18%)	13 (15%)	28 (18%)	8 (17%)	13 (14%)	13 (23%)	7 (18%)	5 (15%)
	HR- HER2+	66 (11%)	11 (14%)	7 (8%)	17 (11%)	4 (9%)	15 (16%)	7 (12%)	4 (10%)	1 (3%)
	HR- HER2-	220 (37%)	16 (21%)	37 (44%)	64 (41%)	23 (49%)	38 (42%)	16 (28%)	14 (36%)	12 (35%)
	Missing	200	21	21	45	18	35	19	25	16
Clinical Tumor Stage										
	cT1	255 (36%)	51 (59%)	25 (26%)	54 (29%)	22 (38%)	22 (19%)	24 (38%)	27 (50%)	30 (65%)
	cT2	314 (44%)	27 (31%)	51 (52%)	92 (49%)	28 (48%)	52 (46%)	32 (50%)	20 (37%)	12 (26%)
	cT3	92 (13%)	5 (6%)	13 (13%)	26 (14%)	5 (9%)	27 (24%)	6 (9%)	6 (11%)	4 (9%)
	cT4	49 (7%)	4 (5%)	9 (9%)	17 (9%)	3 (5%)	13 (11%)	2 (3%)	1 (2%)	0 (0%)
	Missing	77	10	8	14	7	12	12	10	4
Clinical Nodal Stage										
	cN0	442 (62%)	65 (71%)	57 (59%)	113 (61%)	36 (63%)	51 (47%)	44 (67%)	41 (67%)	35 (76%)
	cN1	230 (32%)	23 (25%)	38 (40%)	59 (32%)	19 (33%)	44 (41%)	17 (26%)	20 (33%)	10 (22%)
	cN2	8 (1%)	0 (0%)	1 (1%)	2 (1%)	1 (2%)	2 (2%)	2 (3%)	0 (0%)	0 (0%)
	cN3	31 (4%)	3 (3%)	0 (0%)	12 (6%)	1 (2%)	11 (10%)	3 (5%)	0 (0%)	1 (2%)
	Missing	76	6	10	17	8	18	10	3	4
Clinical Metastases Stage										
	cM0	627 (91%)	85 (99%)	85 (94%)	157 (85%)	47 (89%)	94 (86%)	58 (94%)	55 (98%)	46 (100%)
	cM1	60 (9%)	1 (1%)	5 (6%)	28 (15%)	6 (11%)	15 (14%)	4 (6%)	1 (2%)	0 (0%)
	Missing	100	11	16	18	12	17	14	8	4
Overall Disease Stage										
	Stage 1	142 (19%)	29 (31%)	11 (11%)	25 (13%)	9 (15%)	13 (11%)	19 (26%)	16 (26%)	20 (41%)
	Stage 2	399 (53%)	48 (51%)	61 (61%)	100 (51%)	33 (56%)	66 (56%)	34 (47%)	34 (56%)	23 (47%)
	Stage 3	145 (19%)	16 (17%)	23 (23%)	41 (21%)	11 (19%)	23 (19%)	16 (22%)	9 (15%)	6 (12%)
	Stage 4	63 (8%)	1 (1%)	5 (5%)	29 (15%)	6 (10%)	16 (14%)	4 (5%)	2 (3%)	0 (0%)
	Missing	38	3	6	8	6	8	3	3	1
Pregnancy Outcome										
	Continued	228 (48%)	41 (42%)	69 (65%)	106 (52%)	12 (18%)	NA	NA	NA	NA
	Interrupted	37 (8%)	29 (30%)	1 (1%)	1 (1%)	6 (9%)	NA	NA	NA	NA
	Unknown	206 (44%)	27 (28%)	36 (34%)	96 (47%)	47 (72%)	NA	NA	NA	NA
	Not Applicable	316	NA	NA	NA	NA	126	76	64	50
Time to First Treatment										
	Median (Days)	20	26	17	16	21	22	22	22	25
	Missing	189	17	18	62	15	34	19	18	6
5-Year Overall Survival										
	Survival Probability	78%	91%	72%	68%	76%	69%	85%	90%	92%
	Missing	35	3	5	8	6	9	2	2	0

For each PABC-subgroup, a subdivision is made for each trimester of diagnosis, and the respective intervals after childbirth or after the interrupted pregnancy

*PABC Pregnancy Associated Breast Cancer, PrBC Breast Cancer during Pregnancy, PPBC Postpartum Breast Cancer, AABC Breast Cancer after Interrupted Pregnancy, NST No Specific Type, B&R Bloom & Richardson, ER Estrogen Receptor, PR Progesterone Receptor, HER2 Human Epidermal growth factor Receptor 2, HR Hormone Receptor*

### *PABC (n* = *692) versus non-PABC (n* = *2076)*

Timing of diagnosis for the matched PABC cohort was similarly distributed as for the full cohort, with 408 PrBC (59%), 174 PPBC (25%) and 110 AABC (16%) patients respectively (Table 2). The respective ages at diagnosis were similar between PABC (median 34 years, range 19–48 years) and non-PABC (median 35 years, range 18–49), as cases and controls were matched based on age.

**Table 2:** Comparison of histopathologic and clinical characteristics between the PABC cohort (n = 692) and their 1:3 matched non-PABC comparators (n = 2076). Patients were matched based on age at diagnosis and year of diagnosis

		PABC	Non-PABC	P-value
		PrBC/PPBC/AABC		
		(n = 692)	(n = 2,076)	
		N (%)	N (%)	
Age				
	Median (IQR)	34 years (31–37)	35 years (31–38)	*0.313*^*a*^
	Minimum—Maximum	19–48 years	18–49 years	
Trimester of Diagnosis				
	PrBC Trimester 1	87 (13%)	NA	
	PrBC Trimester 2	93 (13%)	NA	
	PrBC Trimester 3	171 (25%)	NA	
	PrBC Trimester Unknown	57 (8%)	NA	
	PPBC 0–6 Months	109 (16%)	NA	
	PPBC 6–12 Months	65 (9%)	NA	
	AABC 0–6 Months	60 (9%)	NA	
	AABC 6–12 Months	50 (7%)	NA	
Histological Subtype				*0.114*^*b*^
	NST	584 (84%)	1,707 (82%)	
	Lobular	17 (2%)	86 (4%)	
	Other	91 (13%)	283 (14%)	
	Missing	0	0	
B&R Grade				< *0.001*^*b*^
	1	23 (4%)	132 (8%)	
	2	143 (26%)	565 (34%)	
	3	390 (70%)	988 (59%)	
	Missing	136	391	
Estrogen Receptor				< *0.001*^*b*^
	ER- (< 10%)	257 (49%)	620 (39%)	
	ER+ (≥ 10%)	264 (51%)	990 (61%)	
	Missing	171	466	
Progesterone Receptor				< *0.001*^*b*^
	PR- (< 10%)	302 (59%)	755 (48%)	
	PR+ (≥ 10%)	213 (41%)	826 (52%)	
	Missing	177	495	
HER2 Status				*0.311*^*b*^
	HER2-	343 (72%)	1,063 (75%)	
	HER2+	132 (28%)	360 (25%)	
	Missing	217	653	
Intrinsic subtypes				< *0.001*^*b*^
	HR+ HER2-	165 (35%)	661 (47%)	
	HR+ HER2+	76 (16%)	226 (16%)	
	HR- HER2+	51 (11%)	128 (9%)	
	HR- HER2-	174 (37%)	382 (27%)	
	Missing	226	679	
Clinical Tumor Stage				< *0.001*^*b*^
	cT1	242 (37%)	843 (44%)	
	cT2	292 (44%)	829 (43%)	
	cT3	85 (13%)	173 (9%)	
	cT4	41 (6%)	67 (4%)	
	Missing	32	164	
Clinical Nodal Stage				*0.295*^*b*^
	cN0	421 (64%)	1,325 (67%)	
	cN1	206 (31%)	555 (28%)	
	cN2	7 (1%)	15 (1%)	
	cN3	28 (4%)	69 (4%)	
	Missing	30	112	
Clinical Metastases Stage				*0.016*^*b*^
	cM0	586 (91%)	1,807 (94%)	
	cM1	56 (9%)	113 (6%)	
	Missing	50	156	
Overall Disease Stage				< *0.001*^*b*^
	Stage 1	132 (19%)	620 (30%)	
	Stage 2	367 (53%)	1,014 (49%)	
	Stage 3	131 (19%)	317 (15%)	
	Stage 4	58 (8%)	117 (6%)	
	Missing	4	8	
Time to First Treatment				< *0.001*^*a*^
	Median (Days)	20	24	
	Missing	99	284	
5-Year Overall Survival				< *0.001*^*c*^
	Survival Probability	77%	85%	
	Missing	0	0	

^a^Brown-Mood Median Test, ^b^Pearson’s Chi-squared Test, ^c^Logrank Test

*PABC Pregnancy Associated Breast Cancer, PrBC Breast Cancer during Pregnancy, PPBC Postpartum Breast Cancer, AABC Breast Cancer after Interrupted Pregnancy, NA Not Applicable, IQR Interquartile range, NST No Specific Type, B&R Bloom & Richardson, ER Estrogen Receptor, PR Progesterone Receptor, HER2 Human Epidermal growth factor Receptor 2, HR Hormone Receptor*

**Fig. 3:**
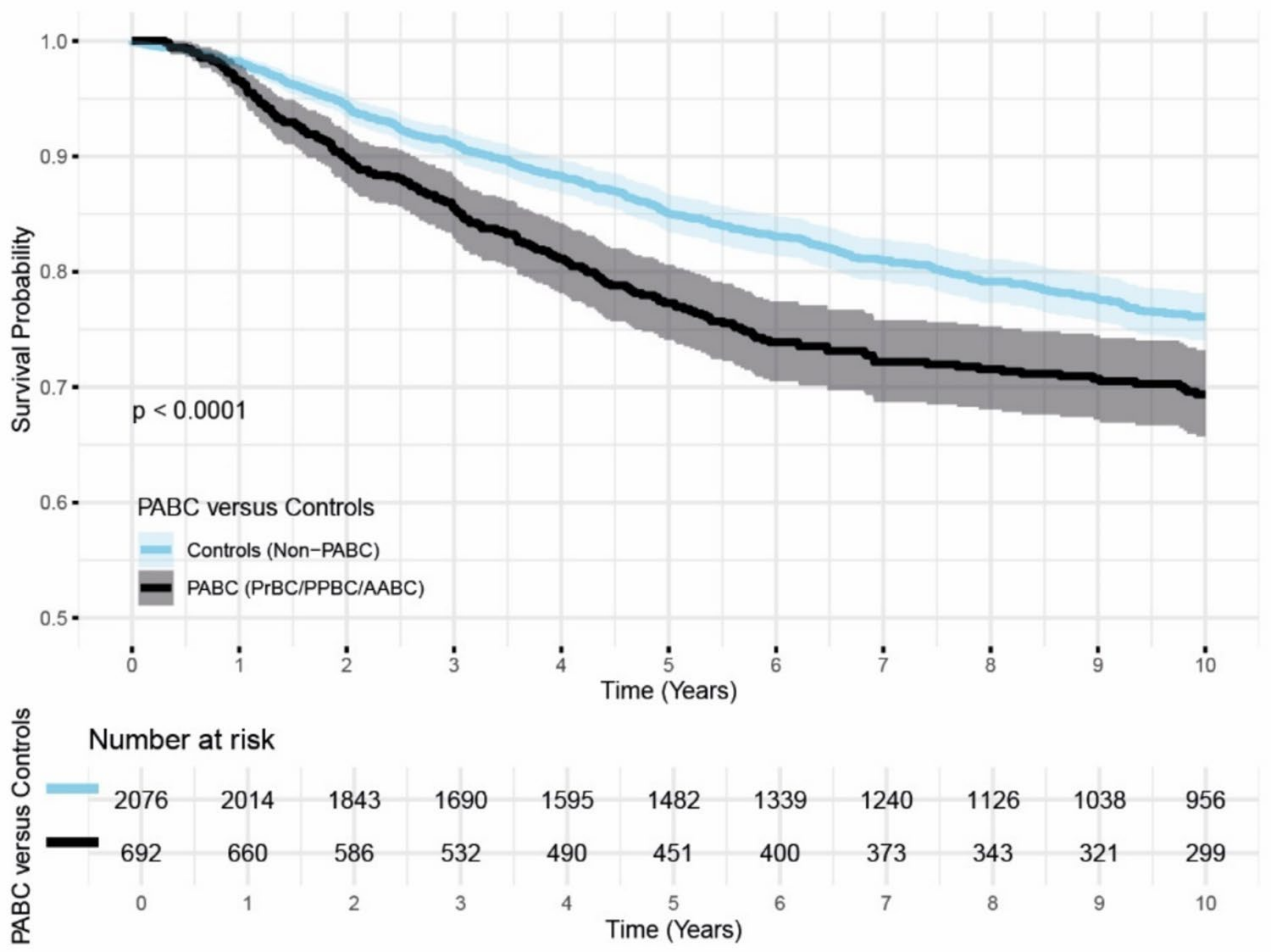
Kaplan–Meier graph depicting the 10-year OS of the PABC cohort (n = 692, in black) and the matched non-PABC comparators (n = 2,076, in blue). Shaded areas represent the respective 95% confidence intervals. P-value calculated with logrank statistics *PABC Pregnancy Associated Breast Cancer, PrBC Breast Cancer during Pregnancy, PPBC Postpartum Breast Cancer, AABC Breast Cancer after Interrupted Pregnancy*

PABC patients more often exhibited aggressive tumor characteristics in comparison to their matched controls, with more frequently grade 3 tumors (70% versus 59%, *P* < 0.001), more frequently triple negative receptor status (ERneg/PRneg/HER2neg; 37% versus 27%, *P* < 0.001) and more advanced disease stages at diagnosis (19% versus 30% stage 1 and 27% versus 21% stage 3/4 at diagnosis, *P* < 0.001) (Table 2). Notably, the interval between diagnosis and first treatment for PABC patients was shorter than for the non-PABC comparators. Importantly, the five-year overall survival was poorer for PABC (77% versus 85%, *P* < 0.001), as also depicted in the Kaplan–Meier graph (Fig. 3).

To exclude potential influence of differences in treatment strategies, we analyzed this in two ways. First, treatment comparisons between PABC and non-PABC were made on the basis of the intrinsic subtypes, which showed similar applied treatment frequencies (Table S1). Next, to evaluate potentially improved treatment protocols in more recent years, we performed a sensitivity analysis with patients from our cohort diagnosed in the most recent ten years (i.e., 2012–2022; n = 275 PABC and n = 825 non-PABC). This analysis yielded similar results for the overall observed differences in histopathologic characteristics and prognosis (Table S2).

Lastly, we performed a multivariable analysis on complete cases (n = 1,538; n = 383 PABC; n = 1155 controls) that were censored after a 10-year follow-up period. The model was corrected for PABC/non-PABC status, histologic grade, intrinsic subtype, overall disease stage, administration of chemotherapy, HER2-targeted therapy, endocrine therapy and surgical procedure performed. Here, PABC patients persisted in their significantly worse survival (HR 1.569, 95% CI 1.056–2.330) in comparison to non-PABC patients. The full output of this model can be found in supplementary table S3.

### PABC: Influence of the trimesters

**Fig. 4:**
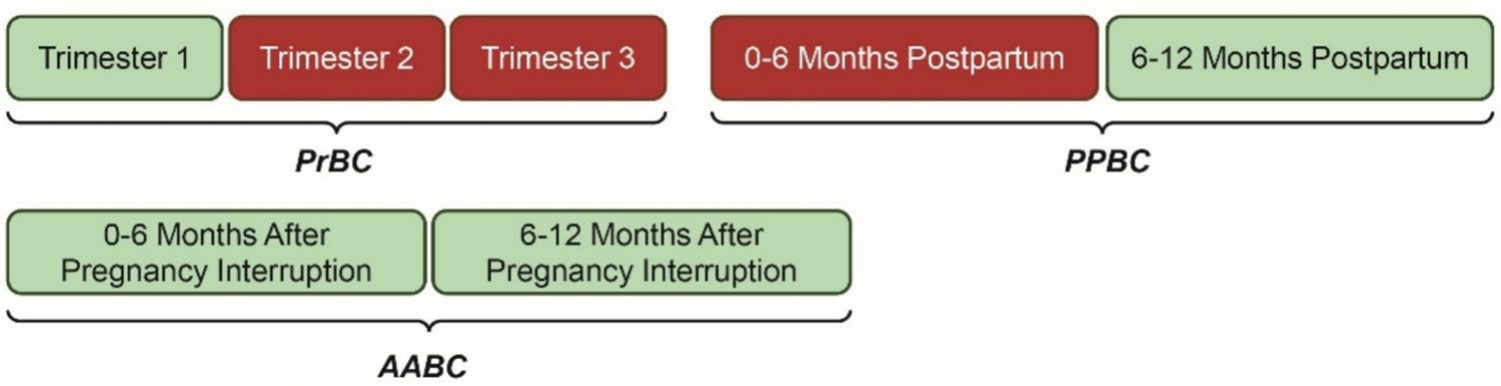
Graphical summary of our findings, displaying the various PABC subgroups (PrBC, PPBC and AABC) and the respective time intervals when patients in our cohort were diagnosed with invasive breast cancer. Diagnostic PABC intervals highlighted in light green are associated with more favorable disease characteristics and have a similar survival in comparison to non-PABC controls. Diagnostic PABC intervals highlighted in red are significantly associated with unfavorable disease characteristics and a significantly worse survival, both in comparison to the favorable PABC subgroups and to non-PABC controls *PrBC Breast Cancer during Pregnancy, PPBC Postpartum Breast Cancer, AABC Breast Cancer after Interrupted Pregnancy*

Within PABC, several differences are observed for the various subgroups (PrBC-trimesters, AABC, PPBC-timeframe) in Table 1. Notably, tumors of patients diagnosed at an advanced stage of pregnancy (i.e., trimesters 2 and 3) or shortly postpartum (< 6 months PPBC) more often exhibited aggressive characteristics (i.e. grade 3 and lack of hormone receptors). In addition, tumors of these patient groups presented at a more advanced stage at diagnosis. Moreover, survival of these patients was markedly impaired in comparison to patients diagnosed in the first trimester of pregnancy, after an interrupted pregnancy or later postpartum (6–12 months PPBC) (Table 1). The most unfavorable 5-year OS probability was observed in the second and third trimesters (72% and 68%, respectively), whereas the survival of first trimester patients (91%) and AABC patients (90% and 92%) was notably better. Unfortunately, the number of complete cases for multivariable analysis (383/787) was too small to obtain hazard ratio estimates per PrBC trimester and PPBC/AABC interval. Figure 4 represents a graphical summary of our per-subgroup results for tumor characteristics and survival.

## Discussion

This extensive update and expansion of our Dutch Nationwide PABC cohort again demonstrates the aggressive disease characteristics of PABC in comparison to non-PABC. Interestingly, these characteristics were not only mainly present in patients diagnosed in the second and third trimesters of pregnancy, but also in patients shortly postpartum. In contrast, patients diagnosed outside this consecutive ‘time-window’ (i.e. after six months postpartum or early in their pregnancy in trimester 1), had more favorable tumor characteristics and a better overall prognosis. The same held true for the newly identified AABC subgroup in this study, which mainly resembles the favorable PABC subgroups and the non-PABC control group.

This update shows that the second trimester is associated with the most aggressive histopathological characteristics, and the third trimester with the most advanced disease stage, which is in line with the Swedish study of Gkekos et al. [20] Our findings for PABC overall also align with previous literature, highlighting enrichment of triple-negative and HER2-positive subtypes, a higher tumor grade, and more advanced stage at diagnosis. [8–13] While some studies suggest a similar prognosis between PABC and non-PABC after adjustment for confounders (e.g., stage, grade, ER-, PR- and HER2 status) [7], our data show that the poor survival in PABC persists even after multivariable correction (Table S3). This points to the potential intrinsic biological drivers, although residual confounding cannot be ruled out.

A clue to further unravel these biological drivers may be present in the differences observed between the trimesters and postpartum intervals of diagnosis, as variations in immune function and hormonal exposure across the trimesters and within the postpartum window may play a role. During pregnancy, an overall shift towards immunotolerance takes place to harbor the semi-allogenic fetus. This is characterized by an upregulation of regulatory T-cells, combined with lower T-, B- and NK-cell levels. [23] Of these, the lower levels of cytotoxic and helper T-cells have been partially attributed to the (reversible) estrogen-mediated thymic involution during pregnancy. [23] Also, later in pregnancy, an increased activity of the complement system is observed supporting the maternal immune defense. [23, 25] The overall increasing immunotolerance observed later in pregnancy could therefore facilitate immune evasion by developing tumor cells. Interesting in this regard is that a recent study showed higher tumor-infiltrating lymphocyte (TIL) levels in first trimester ER/PR positive PrBC in comparison to second- and third trimester PrBC, suggesting a more active anti-tumor immune microenvironment in early pregnancy. [22] However, it must be noted that in this study the histopathological characteristics differed between the trimesters as well, possibly influencing the observed TIL levels, which are known to differ between subtypes. [22, 26]

Hormonal influences may also explain the observed differences in this study. Tumors diagnosed in the first trimester most likely have developed before conception and are therefore minimally influenced by pregnancy-related changes and hormonal levels. In contrast, tumors diagnosed in later trimesters have likely developed during pregnancy. These tumors were exposed to high levels of human chorionic gonadotropin (hCG) during the first trimester, followed by rising levels of estrogen and progesterone later in pregnancy. [24] The higher proportion of ER/PR negative tumors later in pregnancy may be explained by both mechanisms. A study in twenty-five primary breast cancer patients showed that exogenous hCG administration reduced ER and PR expression and proliferative activity without changes in serum estrogen or progesterone levels that may have accounted for this lower expression. [27] This effect may also influence second and third trimester PrBC. Moreover, the coinciding exposure of these tumors to the rising estrogen and progesterone levels during pregnancy may synergistically lead to receptor down signaling on tumor cells, further resulting in lower ER/PR scores on immunohistochemistry. As a 10% expression cutoff for ER/PR positivity is used in the Netherlands, as in many European countries, some tumors with a low expression (1–9%) will be considered ER/PR negative based on this cutoff. [28, 29] Therefore, for some PABC subgroups a lower cutoff (e.g., 1%) for positivity may have to be considered. Future research should evaluate this for the PABC population and its trimester-based subgroups.

Beyond these classical hormone-receptor pathways, estrogen has also been shown to stimulate breast cancer growth through various effects on the tumor microenvironment (TME), even in ER-negative disease. [30, 31] An example of this is the estrogen-induced infiltration of M2 (“pro-tumor”) macrophages that has been indicated in literature. [30] Additionally, pregnancy is also characterized by elevated levels of many other hormones and growth factors, such as prolactin, glucocorticoids, growth hormone and insulin-like growth factor. [32] One can hypothesize that these factors, involved in amongst others fetal development, mammary gland differentiation and lipid metabolism, may also influence tumorigenesis during pregnancy. Moreover, the physiological stress of the pregnancy itself may also modulate tumor biology, as previous research has shown that both exposure to acute and chronic stressors can accelerate breast cancer tumor growth and alter the TME towards a more immunosuppressive state. [33, 34] If this is indeed the case, these factors will more likely influence tumors later in pregnancy and shortly postpartum as well, as these physiological alterations will then have had time (to some extent) to influence tumorigenesis. To further unravel these pathways, future studies characterizing the TME of PABC may shed further light on these precise interactions. Although in-depth molecular genetic studies have been performed within PABC, these lacked sufficient sample sizes to compare all individual PABC subgroups. [35]

Besides the differences in gestational trimesters, we found relevant differences within the PPBC subgroup. Whilst PPBC diagnosed within six months postpartum resembles late-pregnancy tumors, PPBC diagnosed between six and twelve months postpartum shows more favorable tumor characteristics and an improved prognosis. Potentially, tumors diagnosed shortly postpartum may have originated during pregnancy as well, explaining their resemblance to second and third trimester PrBC. A prior meta-analysis also indicated the worst prognosis within PPBC for patients diagnosed < 12 months postpartum, a risk that decreased with larger postpartum intervals but remained significantly higher until six years postpartum. [7] As no subdivision was made in this meta-analysis at six months postpartum, future research may further divide PPBC cohorts into smaller postpartum intervals to study the here observed six-month cutoff further.

In postpartum tumorigenesis, the involution of the breast has been proposed as a key contributor. [36] This process is characterized by apoptosis of mammary epithelial cells, extensive tissue remodeling and long-term alterations in breast immune infiltration. [36, 37] Even so, it has been proposed to exploit involution mechanisms for targeted prevention of breast cancer in the postpartum window. [38, 39] This increased postpartum breast cancer risk is specifically elevated in patients of older age at first pregnancy and may therefore become more and more relevant. [38–40]

The newly described AABC subgroup provides an additional perspective. Although previous research found no evidence of an elevated breast cancer risk after either a spontaneous or induced abortion [41, 42], it was unknown whether patients diagnosed with breast cancer shortly (< 1 year) after a spontaneous or induced abortion exhibit similar disease characteristics to PPBC. It must be noted that the specific pregnancy duration was not known for all AABC cases, although the majority of abortions occurred in the late first or early second trimester (data not presented). Whilst our AABC cohort had a relatively limited sample size (N = 114) for subgroup analyses, patients diagnosed within six months after an interrupted pregnancy (n = 64) showed a tendency towards more aggressive features in comparison to 6–12-month AABC (n = 50). Nevertheless, their survival probability was markedly better (+ 21%) than the 0–6 months PPBC subgroup. This may be explained by the fact that AABC (especially after an interruption in the first or early second trimester), similar to trimester 1 PrBC, has only minimally been exposed to pregnancy-related biological changes. Together, AABC and trimester 1 PrBC may therefore represent a biologically distinct and lower-risk group within PABC that is interesting for further studies, also with regard to more personalized and potentially de-escalated treatment.

Our findings show that the proportion of patients per trimester in a PrBC cohort significantly influences the overall PrBC prognosis, and that the heterogeneity between existing international cohorts may also explain why literature is not unanimous on this. Therefore, future PABC research should consistently report the timing of diagnosis (both by trimester and postpartum interval) to enable more precise subgroup analyses, thereby further aiding in elucidating the mechanisms that drive the differences in disease aggressiveness within PABC. Given the complexity of all physiological changes occurring during pregnancy that may influence breast cancer tumor growth, such insights will render clues for future research, and also underscores the potential of PABC tumor models as a diorama for further insight into breast carcinogenesis in general.

Strengths of this study include the large size of our cohort, the nationwide coverage limiting geographic selection bias, the high quality of the registry data, and the presence of a large matched control group. Moreover, this made it possible to make subdivisions in specific PrBC/PPBC/AABC subgroups and to observe prognostic differences between these subgroups for which the underlying causes are not yet known, which may guide future in-depth PABC-research.

A limitation of our study is the primary identification of cases using pathology reports. This may have impacted the inclusion of PPBC patients, as clinicians are less prone to mention a recent pregnancy instead of a current pregnancy. Moreover, we do not have data on parity status and previous or subsequent pregnancies of the included patients. This also holds true for our matched non-PABC comparators, of whom several might have been pregnant in the five to ten years before their breast cancer diagnosis. Although we did not know the trimester of diagnosis of all PrBC patients, namely those in the “trimester unknown” subgroup, these patients will likely have mainly consisted of second- and third trimester diagnoses, as the pregnancy of these patients was certain which is more likely at a higher gestational age. Last, as multiple tumor-, disease- and survival characteristics were used as endpoints for the statistical analyses in this study, followed by multivariable modelling, there may be a risk for a type I error due to multiplicity of testing. Nevertheless, as the direction of the results (i.e., PABC having more aggressive disease characteristics) remains consistent throughout all analyses, we anticipate that this risk is negligible.

In conclusion, this update reconfirms the more aggressive disease characteristics and worse outcomes for PABC, and particularly identifies patients diagnosed in advanced pregnancy and early postpartum to be most at risk for an unfavorable disease course. Our findings underscore the importance of distinguishing subgroups within PABC by timing of breast cancer diagnosis, and highlight the need for more in-depth mechanistic research into the interplay of hormonal, immunologic, and microenvironmental factors in pregnancy and the development of breast cancer.

## Supplementary Information

Below is the link to the electronic supplementary material.

Supplementary file1 (DOCX 32 KB)

## Data Availability

The dataset used for this study was selected based on selected pseudonymized pathology reports from the Dutch Archive for Cyto- and Histopathology (PALGA), after which these records were linked at patient-level to clinical and outcome data from the Dutch Cancer Registry. The pseudonymized nature of these records prohibits us from sharing this data publicly. Researchers who want to access the data can submit a formal request at PALGA and the Dutch Cancer Registry.
