# Supplementary material for: Worse prognosis for breast cancer diagnosed in advanced pregnancy and shortly postpartum: an update of the Dutch pregnancy-associated breast cancer cohort
Source: Breast Cancer Res Treat. 2025 Aug 12;214(2):191–204. doi: 10.1007/s10549-025-07806-3 (PMC12464139; doi:10.1007/s10549-025-07806-3)
Supplement: Supplementary file 1 — Supplementary file1 (DOCX 32 KB) [file 10549_2025_7806_MOESM1_ESM.docx]

**Supplementary Data
Manuscript: *Worse Prognosis for Breast Cancer diagnosed in Advanced Pregnancy and Shortly Postpartum: An Update of the Dutch Pregnancy-Associated Breast Cancer Cohort***

**Supplementary Table 1:** Comparison of applied treatments per intrinsic subtype for both PABC and their matched non-PABC controls.

**Supplementary Table 2:** Comparison of histopathologic and clinical characteristics between patients diagnosed in the ten most recent years of our cohort (N=1100, years 2012-2022) in the PABC cohort (n=275) and their 1:3 matched non-PABC comparators (n=825).

**Supplementary Table 3:** Output of a multivariable Cox regression model on complete cases (N=1,538) for survival, especially comparing PABC to non-PABC.

**Supplementary Table 1** Comparison of applied treatments per intrinsic subtype for both PABC and their matched non-PABC controls.

|  |  | **HR+HER2+** | | **HR+HER2-** | | **HR- HER2+** | | **HR-HER2-** | |
| --- | --- | --- | --- | --- | --- | --- | --- | --- | --- |
|  |  | Non-PABC (n=226) | PABC (n=76) | Non-PABC (n=661) | PABC (n=165) | Non-PABC (n=128) | PABC (n=51) | Non-PABC (n=382) | PABC (n=174) |
| **Chemotherapy** | |  |  |  |  |  |  |  |  |
|  | Applied | 219 (97%) | 72 (95%) | 519 (79%) | 142 (86%) | 123 (96%) | 51 (100%) | 362 (95%) | 172 (99%) |
|  | Not applied | 7 (3.1%) | 4 (5.3%) | 142 (21%) | 23 (14%) | 5 (3.9%) | 0 (0%) | 20 (5.2%) | 2 (1.1%) |
|  | Missing | 0 | 0 | 0 | 0 | 0 | 0 | 0 | 0 |
| *P-value* | | *0.477 ^a^* | | *0.030 ^a^* | | *0.323 ^a^* | | *0.022 ^b^* | |
| **Endocrine therapy** | |  |  |  |  |  |  |  |  |
|  | Applied | 198 (88%) | 58 (76%) | 529 (80%) | 142 (86%) | 7 (5.5%) | 3 (5.9%) | 21 (5.5%) | 7 (4.0%) |
|  | Not applied | 28 (12%) | 18 (24%) | 132 (20%) | 23 (14%) | 121 (95%) | 48 (94%) | 361 (95%) | 167 (96%) |
|  | Missing | 0 | 0 | 0 | 0 | 0 | 0 | 0 | 0 |
| *P-value* | | *0.018 ^a^* |  | *0.076 ^a^* |  | *>0.999 ^b^* |  | *0.461 ^a^* |  |
| **HER2-targeted therapy** | |  |  |  |  |  |  |  |  |
|  | Applied | 211 (93%) | 70 (92%) | 10 (1.5%) | 3 (1.8%) | 116 (91%) | 49 (96%) | 2 (0.5%) | 1 (0.6%) |
|  | Not applied | 15 (6.6%) | 6 (7.9%) | 651 (98%) | 162 (98%) | 12 (9.4%) | 2 (3.9%) | 380 (99%) | 173 (99%) |
|  | Missing | 0 | 0 | 0 | 0 | 0 | 0 | 0 | 0 |
| *P-value* | | *0.709 ^a^* |  | *0.730 ^b^* |  | *0.355 ^b^* |  | *>0.999 ^b^* |  |
| **Surgical Procedure** | |  |  |  |  |  |  |  |  |
|  | BCS + SNP | 73 (34%) | 20 (29%) | 218 (34%) | 45 (28%) | 43 (36%) | 14 (30%) | 141 (38%) | 53 (32%) |
|  | BCS + ALND | 8 (3.7%) | 3 (4.3%) | 38 (6.0%) | 10 (6.3%) | 6 (5.1%) | 4 (8.5%) | 34 (9.2%) | 9 (5.4%) |
|  | Mastectomy + SNP | 78 (36%) | 26 (38%) | 240 (38%) | 62 (39%) | 35 (30%) | 14 (30%) | 116 (32%) | 55 (33%) |
|  | Mastectomy + ALND | 56 (26%) | 20 (29%) | 135 (21%) | 41 (26%) | 33 (28%) | 15 (32%) | 74 (20%) | 50 (30%) |
|  | Unspecified surgery | 0 (0%) | 0 (0%) | 1 (0.2%) | 0 (0%) | 0 (0%) | 0 (0%) | 3 (0.8%) | 0 (0%) |
|  | No surgery performed | 11 | 7 | 29 | 7 | 10 | 4 | 14 | 7 |
| *P-value* | | *0.608 ^b^* |  | *0.550 ^b^* |  | *0.797 ^b^* |  | *0.051 ^b^* |  |
| **Radiotherapy** | |  |  |  |  |  |  |  |  |
|  | Applied | 137 (61%) | 46 (61%) | 425 (64%) | 118 (72%) | 92 (72%) | 32 (63%) | 252 (66%) | 121 (70%) |
|  | Not applied | 89 (39%) | 30 (39%) | 236 (36%) | 47 (28%) | 36 (28%) | 19 (37%) | 130 (34%) | 53 (30%) |
|  | Missing | 0 | 0 | 0 | 0 | 0 | 0 | 0 | 0 |
| *P-value* | | *0.989 ^a^* |  | *0.080 ^a^* |  | *0.232 ^a^* |  | *0.406 ^a^* |  |

^a^ Pearson’s Chi-squared Test, ^b^ Fisher’s Exact Test

*Abbreviations: PABC: pregnancy-associated breast cancer, HR: hormone receptor, HER2: human epidermal growth factor receptor 2, BCS: breast conserving surgery, SNP: sentinel node procedure, ALND: axillary lymph node dissection.*

**Supplementary Table 2** Comparison of histopathologic and clinical characteristics between patients diagnosed in the ten most recent years of our cohort (N=1100, years 2012-2022) in the PABC cohort (n=275) and their 1:3 matched non-PABC comparators (n=825).

|  |  | **PABC** | **Non-PABC** | **P-value** |
| --- | --- | --- | --- | --- |
|  |  | PrBC/PPBC/AABC |  |  |
|  |  | (n=275) | (n=825) |  |
|  | | N (%) | N (%) |  |
| **Age (Median)** | | 34 | 35 | *0.424 ^a^* |
| **Histologic Subtype** | |  |  | *0.394 ^b^* |
|  | NST | 247 (90%) | 734 (89%) |  |
|  | Lobular | 5 (1.8%) | 28 (3.4%) |  |
|  | Other | 23 (8.4%) | 63 (7.6%) |  |
|  | Missing | 0 | 0 |  |
| **B&R Grade** | |  |  | *<0.001 ^b^* |
|  | 1 | 8 (3.4%) | 65 (9.2%) |  |
|  | 2 | 67 (28%) | 269 (38%) |  |
|  | 3 | 162 (68%) | 369 (52%) |  |
|  | Missing | 38 | 122 |  |
| **Estrogen Receptor** | |  |  | *<0.001 ^b^* |
|  | ER- (<10%) | 129 (47%) | 284 (35%) |  |
|  | ER+ (≥10%) | 145 (53%) | 533 (65%) |  |
|  | Missing | 1 | 8 |  |
| **Progesterone Receptor** | |  |  | *0.013 ^b^* |
|  | PR- (<10%) | 148 (54%) | 368 (45%) |  |
|  | PR+ (≥10%) | 126 (46%) | 448 (55%) |  |
|  | Missing | 1 | 9 |  |
| **HER2 Status** | |  |  | *>0.999 ^b^* |
|  | HER2- | 202 (74%) | 598 (74%) |  |
|  | HER2+ | 72 (26%) | 215 (26%) |  |
|  | Missing | 1 | 12 |  |
| **Intrinsic subtypes** | |  |  | *0.001 ^b^* |
|  | HR+ HER2- | 104 (38%) | 401 (49%) |  |
|  | HR+ HER2+ | 46 (17%) | 140 (17%) |  |
|  | HR- HER2+ | 26 (9.5%) | 74 (9.1%) |  |
|  | HR- HER2- | 98 (36%) | 196 (24%) |  |
|  | Missing | 1 | 14 |  |
| **Clinical Tumor Stage** | |  |  | *0.006 ^b^* |
|  | cT1 | 88 (33%) | 297 (37%) |  |
|  | cT2 | 123 (46%) | 384 (48%) |  |
|  | cT3 | 40 (15%) | 91 (11%) |  |
|  | cT4 | 18 (6.7%) | 21 (2.6%) |  |
|  | Missing | 6 | 32 |  |
| **Clinical Nodal Stage** | |  |  | *0.027 ^c^* |
|  | cN0 | 151 (55%) | 529 (65%) |  |
|  | cN1 | 101 (37%) | 226 (28%) |  |
|  | cN2 | 2 (0.7%) | 7 (0.9%) |  |
|  | cN3 | 20 (7.3%) | 58 (7.1%) |  |
|  | Missing | 1 | 5 |  |
| **Clinical Metastases Stage** | |  |  | *0.238 ^b^* |
|  | cM0 | 249 (91%) | 767 (93%) |  |
|  | cM1 | 26 (9.5%) | 58 (7.0%) |  |
|  | Missing | 0 | 0 |  |
| **Overall Disease Stage** | |  |  | *0.001 ^b^* |
|  | Stage 1 | 50 (18%) | 244 (30%) |  |
|  | Stage 2 | 143 (52%) | 398 (48%) |  |
|  | Stage 3 | 55 (20%) | 122 (15%) |  |
|  | Stage 4 | 26 (9.5%) | 58 (7.1%) |  |
|  | Missing | 1 | 3 |  |
| **Time to First Treatment** | |  |  | *<0.001 ^a^* |
|  | Median (Days) | 21 | 28 |  |
| **5-Year Overall Survival** | |  |  | *<0.001 ^d^* |
|  | Survival Probability | 83% | 90% |  |
|  | Missing | 0 | 0 |  |

^a^ Brown-Mood Median Test, ^b^ Pearson’s Chi-squared Test, ^c^ Fisher’s Exact Test, ^d^ Logrank Test

Patients were matched based on age at diagnosis and year of diagnosis.

*Abbreviations: PABC: Pregnancy Associated Breast Cancer, PrBC: Breast Cancer during Pregnancy, PPBC: Postpartum Breast Cancer, AABC: Breast Cancer after Interrupted Pregnancy, NST: No Specific Type, B&R: Bloom & Richardson, ER: Estrogen Receptor, PR: Progesterone Receptor, HER2: Human Epidermal growth factor Receptor 2, HR: Hormone Receptor.*

**Supplementary Table 3** Output of a multivariable Cox regression model on complete cases (N=1,538) for survival, especially comparing PABC to non-PABC.

|  | | **Patients (n)** | **Events (n, %)** | **Hazard Ratio** | **Lower 95% CI** | **Upper 95% CI** | **P-value** |
| --- | --- | --- | --- | --- | --- | --- | --- |
| **Subgroup** | |  |  |  |  |  |  |
|  | Non-PABC | 1155 | 129 (11%) | *1.0 (Ref)* | *NA* | *NA* | *NA* |
|  | PABC | 383 | 67 (17%) | **1.569** | **1.056** | **2.330** | **0.025** |
| **Grade** | |  |  |  |  |  |  |
|  | Grade 1 | 113 | 9 (8%) | *1.0 (Ref)* | *NA* | *NA* | *NA* |
|  | Grade 2 | 510 | 52 (10%) | 1.668 | 0.544 | 5.116 | 0.371 |
|  | Grade 3 | 915 | 135 (15%) | 1.422 | 0.474 | 4.264 | 0.530 |
| **Intrinsic Subtype** | |  |  |  |  |  |  |
|  | HR+HER2- | 702 | 80 (11%) | *1.0 (Ref)* | *NA* | *NA* | *NA* |
|  | HR+HER2+ | 240 | 20 (8%) | 0.897 | 0.229 | 3.514 | 0.876 |
|  | HR-HER2+ | 136 | 15 (11%) | 0.961 | 0.237 | 3.895 | 0.956 |
|  | HR-HER2- | 460 | 81 (18%) | 0.976 | 0.445 | 2.142 | 0.952 |
| **Disease Stage** | |  |  |  |  |  |  |
|  | Stage I | 459 | 22 (5) | *1.0 (Ref)* | *NA* | *NA* | *NA* |
|  | Stage II | 788 | 87 (11%) | **1.936** | **1.021** | **3.672** | **0.043** |
|  | Stage III | 248 | 61 (25%) | **3.324** | **1.542** | **7.163** | **0.002** |
|  | Stage IV | 43 | 26 (60%) | **42.596** | **8.635** | **210.121** | **<0.001** |
| **Chemotherapy** | |  |  |  |  |  |  |
|  | Not applied | 163 | 18 (11%) | *1.0 (Ref)* | *NA* | *NA* | *NA* |
|  | Applied | 1,375 | 178 (13%) | 1.138 | 0.454 | 2.854 | 0.782 |
| **HER2-targeted therapy** | |  |  |  |  |  |  |
|  | Not applied | 1,175 | 164 (14%) | *1.0 (Ref)* | *NA* | *NA* | *NA* |
|  | Applied | 363 | 32 (9%) | 0.532 | 0.143 | 1.981 | 0.347 |
| **Endocrine therapy** | |  |  |  |  |  |  |
|  | Not applied | 731 | 111 (15%) | *1.0 (Ref)* | *NA* | *NA* | *NA* |
|  | Applied | 807 | 85 (11%) | 0.748 | 0.364 | 1.536 | 0.429 |
| **Surgical Procedure** | |  |  |  |  |  |  |
|  | BCS + SNP | 549 | 30 (6%) | *1.0 (Ref)* | *NA* | *NA* | *NA* |
|  | BCS + ALND | 92 | 17 (18%) | **3.476** | **1.308** | **9.234** | **0.012** |
|  | Mastectomy + SNP | 559 | 57 (10%) | **2.210** | **1.211** | **4.033** | **0.010** |
|  | Mastectomy + ALND | 338 | 92 (27%) | **2.635** | **1.375** | **5.047** | **0.003** |

*Abbreviations: CI: Confidence Interval, Ref: Reference group, PABC: Pregnancy-Associated Breast Cancer, HR: Hormone Receptor, HER2: Human Epidermal growth factor Receptor 2, BCS: Breast Conserving Surgery, SNP: Sentinel Node Procedure, ALND: Axillary Lymph Node Dissection.*
